# Editorial: introduction to the special issue “methodological challenges of complex latent mediator and moderator models”

**DOI:** 10.3758/s13428-026-02985-3

**Published:** 2026-04-17

**Authors:** Julien P. Irmer, Karin Schermelleh-Engel, Peter Schmidt

**Affiliations:** 1 Institute of Psychology, Albert Ludwigs University Freiburg, Engelbergerstrasse 41, 79085 Freiburg im Breisgau, Germany; 2 Institute of Psychology, Goethe University Frankfurt, Frankfurt, Germany; 3 Centre of International Development and Environmental Research (ZEU), University of Giessen, Giessen, Germany

This special issue “Methodological Challenges of Complex Latent Mediator and Moderator Models” addresses the challenges arising from complex latent models designed to capture differences in effects across individuals, groups, or contexts, that is, effects that vary depending on person-specific, situational, or structural conditions and are often formalized using complex latent nonlinear models. In many substantive research questions, effects are therefore not assumed to be universal, but are hypothesized to depend on individual characteristics, situational contexts, or structural conditions. Such research questions can be translated into moderation hypotheses, which formally express that the magnitude or direction of an effect depends on the level or context of another variable. Accordingly, moderated, mediated, and combined moderated mediation models have become central tools for studying such complex relationships.

When latent variables are involved, structural equation modeling (SEM) provides a state-of-the-art framework for separating measurement from structural relations and correcting for measurement errors in the structural relations, including the estimation of conditional and context-specific effects. However, the formal modeling of such dependencies—commonly implemented via nonlinear relationships such as, e.g., interactions, quadratic effects, nonlinear growth, moderated mediation, group-specific effects, context-specific effects or non-continuous outcomes in latent variable models—remains technically demanding. Furthermore, review articles of newly developed methods for applied researchers that also contain the code for replicating the models are not sufficiently often provided. Applied researchers therefore mostly use linear SEM to test the additive components of their models, but revert to manifest, regression-based approaches (e.g., moderated regression or moderated multilevel analysis), when interactions or nonlinearities are present, that is, at a stage when person-specific heterogeneity or contextual dependence becomes substantively central, thereby forfeiting the advantages of latent variable modeling precisely when measurement error and structural complexity are most consequential (Cheung et al., 2021; Cortina et al., 2021; Pokropek et al., 2025).

At the same time, methodological research on latent models for conditional and context-dependent effects including nonlinear latent models has expanded rapidly, with new estimation strategies, diagnostic tools, simulation-based evaluations, and software implementations emerging across different traditions. Therefore, applied researchers can easily lose track of the newest developments, improvements, or recommendations. This special issue responds to the growing need for guidance by assembling 11 contributions that address current methodological challenges in modeling heterogeneity, moderation, and conditional effects in latent variable frameworks, including nonlinear SEM, multilevel, and Bayesian approaches, that evaluate alternative modeling approaches, use Monte Carlo simulation studies and empirical applications, and provide recommendations for practice. Using cross-sectional, longitudinal, multilevel, time-series, or count data sets, the articles not only illustrate the variety of methodological challenges associated with nonlinear relationships that arise in behavioral and social science research, but also offer solutions to these problems. For all empirical analyses and Monte Carlo simulation studies, syntax and data are openly accessible both for replication purposes and transfer to own data. Across these contributions, moderation is understood in a broad sense as the dependence of an effect on the level or context of another variable, whether observed or latent, continuous or categorical, thereby providing a formal framework for modeling heterogeneity in effects across persons, groups, time, or contexts using a diverse set of modeling strategies. The contributions approach these questions using different methodological traditions and data structures, which we organize into four broad subject areas reflecting distinct modeling frameworks rather than distinct substantive questions.

## Overview of the articles

A wide range of methodological approaches to modeling conditional effects and effect variation is covered by the articles of this special issue. These topics include planning and determining the required sample size for studies with nonlinear effects; testing for misspecification in the measurement model as a necessary prerequisite for model testing; derivations and testing of new modeling strategies for nonlinear and context-dependent effects across individuals, clusters, or time; selecting the most suitable model; and comparing different estimation methods for the particular research question. The core characteristics of the contributions to this special issue are presented in Table 1.

**Table 1:** Mapping special issue contributions to core characteristics

Article	Core methodological contribution	Approaches	No. of moderator/quadratic terms	Key results
**Nonlinear SEM**				
Irmer, Klein, and Schermelleh-Engel (2024a)	Development of MSPE for power of a broad class of estimators with exemplification of linear SEM and complex NLSEM	MSPE; ML and MLR; UPI	1/2	MSPE, a general simulation-based framework for calculating the required sample size given power, outperforms alternative approaches under normality and nonnormality
Irmer, Klein, and Schermelleh-Engel (2024b)	Development of an adaptive algorithm for power curve exploration implemented in the R-package powerNLSEM for accurate power estimates for linear SEM and NLSEM	MSPE; LMS, UPI, FSR, SR	1/2	Simulation-based; powerNLSEM using the adaptive approach performs well in terms of bias and Type I error rates for manifest as well as latent approaches
Navarro and Schermelleh-Engel (2025)	Consequences of omitted cross-loadings in NLSEM	Linear SEM with modification indices and Set-ESEM for misspecification detection; LMS	1/2	Misspecification in the measurement model affects especially the nonlinear structure of NLSEM; bias spreads through biased factor covariances to nonlinear relationships
Rosseel, Burghgraeve, Loh, and Schermelleh-Engel (2025)	Development of nonlinear local structural after measurement (LSAM) with implementation in lavaan; tutorial	Nonlinear LSAM vs. three SAM approaches, LMS, and UPI with utilization of “bounded estimation”	3/6	Two-step approach; nonlinear LSAM with several interaction and quadratic effects, nonnormal data, and small sample sizes performs well in terms of bias and MSE in correctly specified quadratic and interaction models
**Moderated mediation**				
Muench and Koch (2025)	Moderated mediation with measurement and structural invariance across continuous moderator variable	IPCR vs. Bayesian MNLFA	1/-	IPCR is fast and flexible, but provides biased parameter estimates for medium to large effect sizes; BMNLFA generally displays less bias, but it is computationally intensive and requires careful selection of prior distributions
Schamberger, Schuberth, and Henseler (2026)	Combination of moderated mediation with Henseler-Ogasawara (H–O) specification	Composite moderated structural equations (CMS) vs. PLSc	1/-	3-step procedure; CMS performs well for correctly specified models with formative and reflective indicators comparable to PLSc in terms of bias and efficiency
**Count LGCM and count regression**				
Bendler and Reinecke (2025)	Moderation in LGCMs via multiple-group analysis with count outcomes; tutorial	Multiple-group LGCM, Bayesian MCMC	1/-	Multiple-group Bayesian LGCMs provide an accessible and precise strategy for examining group differences without requiring complex interaction specifications
Kiefer, Wilker, and Mayer (2024)	Latent variable count regression model with interactions among both latent and manifest covariates	LV-CRM vs GLM-based Poisson or negative binomial regressions; marginal ML	3/-	LV-CRM provides unbiased estimates and interpretable parameters, GLM can yield severely biased interaction estimates
**Multilevel SEM**				
Cox and Kelcey (2025)	Within-, between-, and cross-level latent interactions in multilevel SEMs and partially nested SEMs with latent moderated mediation	SAM vs. Bayesian approaches	3/-	SAM-Croon consistently outperforms Bayesian methods in cross-level interactions and partially nested SEMs, while both methods perform adequately for within-level interactions
Holtmann and Koslowski (2025)	Extension of multilevel latent VAR with latent interaction effects at the dynamic within-person level; tutorial	Bayesian MCMC	4/-	Multilevel latent VAR models with within-level latent interaction effects perform well with reasonable sample sizes for complex models
Humberg, Grund, and Nestler (2024)	Nonlinear effects of random slopes in multilevel SEM including U-shaped, moderation, and congruence effects between within-person associations and between-level outcomes	Multilevel SEM vs. two-step, SI, and PV; Bayesian MCMC	1/-	Nonlinear multilevel SEM performs very well; SI is an alternative when the data are balanced and the level-2 sample size is small

*CMS* composite moderated structural equations, *ESEM* exploratory structural equation modeling, *FSR* factor score regression, *GLM* generalized linear model, *IPCR* individual parameter contribution regression, *LMS* latent moderated structural equations, *LSAM* local SAM, *MLR* robust maximum likelihood estimator, *MNLFA* moderated nonlinear latent factor analysis, *MSPE* model-implied simulation-based power estimation, *NLSEM* nonlinear structural equation modeling, *PLSc* consistent partial least-squares, *LGCM* latent growth curve model, *MCMC* Markov chain Monte Carlo, *LV-CRM* latent variable count regression model, *SAM* structural-after-measurement, *SAM-Croon* SAM with a Croon-based correction, *Set-ESEM* ESEM using predefined sets, *SI* single-indicator approach, *SR* scale regression, *PV* plausible values approach, *UPI* unconstrained product-indicator approach, *VAR* vector-autoregressive model

In the following, the contributions are shortly outlined. We have assigned them to the following subject areas, as listed in Table 1: nonlinear SEM, moderated mediation, count LGCM, and count regression, and multilevel SEM.

### Nonlinear SEM

Irmer et al. (2024a) emphasize the importance of determining adequate sample sizes for nonlinear SEM prior to data collection. Although simulation-based power estimation can, in principle, be applied in almost all scenarios in which data can be generated from a specified model, a general framework for deriving required sample sizes for a given target power has been lacking. The authors propose a novel model-implied simulation-based power estimation (MSPE) method for the *z*-test that exploits the asymptotic normality of parameter estimates from a broad class of estimators. Furthermore, they provide a formal theoretical justification for the approach. MSPE characterizes the relationship between power and sample size as a continuous function by fitting a parametric model.

Extensive Monte Carlo simulations on linear SEM and quadratic and interaction SEM (QISEM) demonstrate that MSPE yields accurate and unbiased power estimates across a wide range of correctly specified and distributionally misspecified scenarios. Moreover, MSPE outperforms alternative strategies, such as the naïve approach of choosing a discrete selection of sample sizes with linear power interpolation or simple logistic regression approaches. These approaches were once considered state-of-the-art and were frequently used. As a broadly applicable power analysis framework not limited to nonlinear SEM, MSPE offers a valuable tool for study planning across diverse modeling contexts and estimator classes.

Building directly on this framework, Irmer et al. (2024b) propose an adaptive algorithm that explores the model-implied power curve and allows for automation of the whole MSPE procedure. They introduce and evaluate the powerNLSEM R package, which implements the adaptive algorithm for MSPE for power estimation in QISEM (Irmer et al., 2024a). After outlining the MSPE approach and presenting a new adaptive algorithm that automatically selects informative sample sizes for efficient power estimation, the authors provide a step-by-step tutorial illustrating how to conduct MSPE using the powerNLSEM R package. Two Monte Carlo simulation studies then demonstrate sample size requirements for latent moderation and moderated mediation models using latent moderated structural equations (LMS; Klein & Moosbrugger, 2000), the unconstrained product-indicator approach (UPI; Kelava & Brandt, 2009; Marsh et al., 2004), factor score regression (FSR; Ng & Chan, 2020), and scale regression (SR).

The results show that powerNLSEM provides accurate power estimates and highlights meaningful differences in performance across estimation methods. The primary contribution of this article is the adaptive algorithm which allows resource-friendly simulation-based power estimation and a practical, user-friendly tool that translates recent methodological advances in power analysis for nonlinear SEM into an accessible workflow for applied researchers.

Navarro and Schermelleh-Engel (2025) investigate the consequences of measurement model misspecification in QISEM, focusing specifically on omitted cross-loadings. Using both an empirical example and a supporting Monte Carlo simulation study, the authors demonstrate systematic patterns of bias arising from such misspecifications. In particular, factor loadings and subsequently factor correlations are biased, and this factor correlation bias spreads to the variance of the latent moderator term as well as to all nonlinear relationships involving moderator and quadratic terms. As a result, nonlinear effects are more strongly affected by omitted cross-loadings than linear effects, potentially altering the functional form of relationships between latent variables and leading to erroneous substantive conclusions.

These findings carry important implications for applied research. While common methods for estimating QISEM, such as LMS (Klein & Moosbrugger, 2000) or UPI (Kelava & Brandt, 2009; Marsh et al., 2004), may yield unbiased estimates under ideal measurement conditions, they provide limited diagnostic tools for detecting unknown measurement misspecifications. The authors therefore recommend systematically evaluating the linear measurement model prior to estimating nonlinear structural effects. Linear SEM fit indices, modification indices, or Set-ESEM (Marsh et al., 2014, 2020), a variant of exploratory structural equation modeling (ESEM; Asparouhov & Muthén, 2009) where factors are grouped into predefined sets allowing cross-loadings only within these sets, can be used to identify potential misspecifications in the linear component, which is typically separable from the nonlinear structural part of the model. Careful measurement model validation should thus be regarded as a prerequisite before estimating nonlinear structural effects.

Rosseel et al. (2025) address the challenge of estimating quadratic and interaction effects in QISEM when models include a large number of nonlinear terms. Established approaches such as LMS (Klein & Moosbrugger, 2000) or UPI (Kelava & Brandt, 2009; Marsh et al., 2004) generally perform well only when the number of nonlinear effects is relatively small also in comparison to sample size. To overcome this limitation, the authors propose nonlinear LSAM, a new method designed for high-complexity nonlinear SEMs. It builds on the recently introduced local structural-after-measurement (local SAM; Rosseel & Loh, 2024) framework. This framework consists of two steps—just as the name suggests. In the first step, the measurement part of the model is estimated, and in the second step, given the estimated measurement model, the structural part of the model is estimated, which improves estimation stability without introducing strong additional assumptions. An empirical application demonstrates the feasibility of the method by estimating a complex model with seven interaction effects simultaneously without convergence problems.

In a Monte Carlo simulation study of a QISEM with an interaction and two quadratic terms, nonlinear LSAM is compared with LMS (Klein & Moosbrugger, 2000), UPI (Kelava & Brandt, 2009; Marsh et al., 2004), and three SAM-based alternatives, i.e., the two-stage method of moments (Wall & Amemiya, 2000, 2003), extensions of the bias-avoiding factor score approach (Ng & Chan, 2020), and Croon-type method for latent interactions (Cox & Kelcey, 2021; Croon, 2002). The results show that nonlinear LSAM provides stable and efficient estimates comparable to SAM-based approaches, even with nonnormal and low-reliability indicators. Overall, the results indicate that nonlinear LSAM offers a robust and scalable solution for estimating highly complex nonlinear SEMs and represents a practical extension of the SAM framework implemented in the *lavaan* package (Rosseel, 2012).

### Moderated mediation

Muench and Koch (2025) investigate the methodological challenges of testing measurement and structural invariance in latent moderated mediation models, particularly when moderation occurs along a continuous covariate. Although violations of measurement invariance can bias conclusions about moderated direct and indirect effects, such invariance is rarely tested with respect to the moderator itself. The authors compare two approaches that allow for the simultaneous assessment of measurement and structural invariance: individual parameter contribution regression (IPCR; Arnold et al., 2019, 2021), a multi-step maximum likelihood procedure, and moderated nonlinear latent factor analysis (MNLFA; Bauer, 2017; Bauer & Hussong, 2009), a one-step model estimated in a Bayesian framework, enabling model evaluation via posterior predictive checks and leave-one-out cross-validation (LOO-CV; Vehtari et al., 2017).

Using an empirical application based on data from the German Family Panel (Brüderl et al., 2022) and a complementary Monte Carlo simulation study, the authors evaluate both approaches with respect to parameter bias and practical usability by varying sample size and effect size. The results indicate that both approaches can recover moderated mediation effects and detect violations of invariance, but they differ in their sensitivity to parameter heterogeneity and in their practical trade-offs between flexibility, computational complexity, and interpretability. Overall, the article contributes a systematic comparison of the two complementary methods IPCR and Bayesian MNLFA and provides clear recommendations for applied researchers on choosing between both methods when analyzing latent moderated mediation models: IPCR is especially well suited for exploratory analysis and MNLFA for confirmatory analysis.

Schamberger et al. (2026) address the inability of existing approaches to moderated mediation to flexibly model unknown-weight composites as mediators and moderators within a full SEM framework. While models involving reflectively measured latent variables are well established, corresponding methods for formative constructs or composites are missing. The authors propose a new approach, the composite moderated structural equations (CMS)**,** that integrates LMS (Klein & Moosbrugger, 2000) with the Henseler–Ogasawara (H–O) specification for composites (Schuberth, 2023). CMS enables researchers to estimate moderated mediation models that include unknown-weight composites while retaining key advantages of SEM. The approach follows a structured three-step procedure in which composite loadings are first estimated, composite scores are then derived, and the moderated mediation model is finally estimated using LMS.

A Monte Carlo simulation study compares CMS with consistent partial least squares (PLSc; Dijkstra & Schermelleh-Engel, 2014), the only alternative approach currently capable of handling moderated mediation with composites in open-source software. The results show that CMS performs well, comparably to PLSc in terms of bias and efficiency. Overall, the article introduces CMS as a theoretically grounded and practically feasible extension of nonlinear SEM for models involving composites and thereby substantially broadens the applicability of complex latent mediator and moderator models.

### Count LGCM and count regression

Bendler and Reinecke (2025) present a tutorial on analyzing categorical moderation effects in latent growth curve models (LGCMs) with count-distributed outcomes using Bayesian multiple-group modeling. While moderation in LGCMs is often implemented via latent interaction terms, such approaches quickly become computationally demanding and difficult to specify when models include nonlinear growth functions, non-Gaussian outcomes, or multiple latent variables. As an alternative, the authors advocate multiple-group comparisons (Jöreskog, 1971; Sörbom, 1974) as conceptually simple and flexible.

Focusing on count data modeled via the negative binomial distribution, the tutorial demonstrates how Bayesian estimation can alleviate key limitations of maximum likelihood approaches. Using data from a large-scale longitudinal study on juvenile delinquency (Boers et al., 2010), the authors illustrate multiple-group LGCMs for unconditional and conditional growth models (Bollen & Curran, 2006) and examine moderation by gender and school type. Bayesian estimation via MCMC (Asparouhov & Muthén, 2010; Depaoli et al., 2023) allows for stable estimation of complex models, facilitates the testing of parameter constraints using Bayesian Wald tests, and supports intuitive interpretation. Overall, the article provides applied researchers with a clear and accessible workflow for conducting moderation analyses in nonlinear LGCMs with count outcomes. By combining multiple-group modeling with Bayesian estimation, the contribution highlights a practical alternative to latent interaction modeling and directly addresses methodological challenges associated with complex mediator and moderator models in longitudinal research.

Kiefer et al. (2024) address the challenge of modeling interactions between latent variables in count regression models. In applied research, count outcomes are often analyzed using generalized linear models (GLMs; e.g., Nelder & Wedderburn, 1972) with observed predictors, implicitly assuming error-free measurement. When predictors are fallible and interactions are of interest, this practice can lead to severe bias, even when predictor reliabilities are high. To overcome these limitations, the authors propose a latent variable count regression model (LV-CRM) allowing for latent covariates as well as interactions among both latent and manifest covariates within a structural equation modeling framework.

The proposed model is formulated as an extension of GLMs that also builds on the generalized structural equation model framework (Rockwood, 2021) and accommodates common count distributions such as Poisson and negative binomial regression. Three Monte Carlo simulation studies compare the LV-CRM with conventional GLM-based approaches, demonstrating that GLM estimates can be substantially biased, whereas the LV-CRM yields largely unbiased regression coefficients and acceptable inferential performance under moderate sample sizes. An applied example from clinical psychology illustrates the practical use of the proposed model. Overall, the article provides an important extension of latent interaction modeling to non-continuous outcomes and highlights the necessity of accounting for measurement error when analyzing interactions in count data.

### Multilevel SEM

Cox and Kelcey (2025) provide a systematic comparison of SAM (Rosseel & Loh, 2024; Rosseel et al., 2025) and Bayesian methods (Asparouhov & Muthén, 2021; Cox & Kelcey, 2021; Smid & Rosseel, 2020) for multilevel SEMs with latent interactions under complex data structures. Although both approaches have been proposed as alternatives to conventional full-information estimators, direct comparisons have been largely limited to simple settings. The authors address this gap by examining estimator performance in multilevel SEMs with within-level, between-level, and cross-level latent interactions, as well as in partially nested SEMs with latent moderated mediation.

A series of Monte Carlo simulation studies shows that estimator performance strongly depends on the type of latent interaction and data structure. SAM approaches perform consistently well across a wide range of conditions. Especially SAM-Croon, a local SAM estimator that utilizes factor score regression with a Croon-based (Croon, 2002) correction (Cox et al., 2023; Rosseel et al., 2025), consistently outperforms Bayesian methods with and without informative priors in cross-level interactions and partially nested SEMs, while both methods perform adequately well for within-level interactions. An additional Monte Carlo simulation study illustrates the flexibility of SAM approaches by extending them to latent moderated mediation models in partially nested data. Overall, the results highlight SAM methods as a versatile and adaptable alternative or complement to Bayesian estimation for nonlinear SEMs with complex data structures. The authors provide recommendations for estimator choice based on model complexity and interaction type.

Holtmann and Koslowski (2025) extend multilevel latent time-series modeling by introducing within-level latent interaction effects into multilevel vector-autoregressive (VAR) models (e.g., Asparouhov et al., 2018; Jongerling et al., 2015). Although multilevel VAR and dynamic structural equation models (Adolf et al., 2017; Kelava & Brandt, 2019) are increasingly used to study within-person dynamics in intensive longitudinal data, applications have typically assumed that dynamic relations are invariant across time and contexts. The authors address this limitation by allowing time-varying latent moderators to influence autoregressive and cross-lagged effects at the within-person level. The proposed models build on latent person-mean centering (Asparouhov et al., 2018; Asparouhov & Muthén, 2019) and explicitly account for measurement error in both predictors and moderators. Using Bayesian estimation via MCMC methods, the authors provide a detailed tutorial and illustrate the approach with two empirical applications examining the temporal dynamics of negative affect, rumination, and mindful attention.

A comprehensive Monte Carlo simulation study evaluates model performance across varying numbers of persons, time points, and model complexity, yielding practical recommendations for applied researchers: Required sample sizes range between at least 25 time points for 50 persons in the simplest fixed-effects models and at least 100 time points for at least 100 persons in random-effects factor models. Overall, the article substantially broadens the scope of latent interaction modeling by integrating moderation into dynamic within-person processes and offering an accessible implementation for complex longitudinal data.

Humberg et al. (2024) investigate the estimation of nonlinear effects of random slopes in multilevel SEM. In many psychological applications, random slopes represent within-person associations (WPAs) between time-varying variables (e.g., Asparouhov et al., 2018; McNeish & Hamaker, 2019), and substantive theories often imply nonlinear relationships between these slopes and person-level outcomes. The authors focus on three common classes of nonlinear hypotheses: U-shaped effects, moderation effects between random slopes, and congruence effects.

A Monte Carlo simulation study evaluates a fully latent multilevel SEM approach for estimating these nonlinear effects and compares it with three simpler alternatives: a conventional two-step approach (Cortina et al., 2021), a plausible-values approach (Mislevy, 1991; von Davier et al., 2009), and a single-indicator (SI) approach (Bollen, 1989; Hayduk, 1987), in which latent variables are defined via single-indicator measurement models for the estimated random slopes and then used as predictors in a single-level SEM. The results show that the latent multilevel SEM approach yields good performance in terms of accuracy of parameter estimates, coverage rates, and likelihood of correctly detecting or rejecting nonlinear effects. The SI approach qualifies as a viable alternative to multilevel SEM, especially when only a small number of level-2 units are available, whereas the simple two-step and plausible-values approaches exhibit notable biases or reduced inferential accuracy. An empirical data set with combined data from two daily diary studies illustrates the estimation of nonlinear effects of WPAs.

## Conclusion

The contributions in this special issue collectively reflect the breadth of current methodological developments for modeling conditional and context-dependent effects with latent variables and highlight the practical challenges that arise when studying complex mediated and moderated processes with latent variables. While the articles differ in their focus, ranging from power analysis, diagnostics, and estimation strategies to mixture, generalized, and multilevel modeling, they converge in demonstrating that variation across persons, groups, or contexts with latent variables requires careful specification, evaluation, and often simulation-based planning, particularly when nonlinear components are involved. The tools, comparisons, and guidelines provided here aim to support applied researchers in selecting appropriate methods and in selecting and implementing appropriate latent variable modeling strategies for studying conditional and moderated effects in substantive research. Additionally, the availability of computer codes and simulation workflows used in these articles enables interested researchers to transfer them to their own applications and data sets more easily.

The editors hope that this special issue not only documents the newest methodological developments but also serves as a practical resource, enabling researchers to more confidently address moderation and effect heterogeneity using appropriate latent variable models in their applied work.

## Data Availability

Not applicable.

## References

[CR1] Adolf, J. K., Voelkle, M. C., Brose, A., & Schmiedek, F. (2017). Capturing context-related change in emotional dynamics via fixed moderated time series analysis. *Multivariate Behavioral Research,**52*(4), 499–531. 10.1080/00273171.2017.1321978 28532179 10.1080/00273171.2017.1321978

[CR2] Arnold, M., Oberski, D. L., Brandmaier, A. M., & Voelkle, M. C. (2019). Identifying heterogeneity in dynamic panel models with individual parameter contribution regression. *Structural Equation Modeling: A Multidisciplinary Journal,**27*, 613–628. 10.1080/10705511.2019.1667240

[CR3] Arnold, M., Brandmaier, A. M., & Voelkle, M. C. (2021). Predicting differences in model parameters with individual parameter contribution regression using the R package ipcr. *Psych,**3*(3), 360–385. 10.3390/psych3030027

[CR4] Asparouhov, T., & Muthén, B. (2009). Exploratory structural equation modeling. *Structural Equation Modeling: A Multidisciplinary Journal,**16*(3), 397–438. 10.1080/10705510903008204

[CR5] Asparouhov, T., & Muthén, B. (2021). Bayesian estimation of single and multilevel models with latent variable interactions. *Structural Equation Modeling: A Multidisciplinary Journal,**28*(2), 314–328. 10.1080/10705511.2020.1761808

[CR6] Asparouhov, T., Hamaker, E. L., & Muthén, B. (2018). Dynamic structural equation models. *Structural Equation Modeling: A Multidisciplinary Journal,**25*(3), 359–388. 10.1080/10705511.2017.1406803

[CR7] Asparouhov, T., & Muthén, B. (2010). *Bayesian analysis using Mplus: Technical implementation version 3* (Tech. Rep.). http://statmodel.com/download/Bayes3.pdf

[CR8] Bauer, D. J. (2017). A more general model for testing measurement invariance and differential item functioning. *Psychological Methods,**22*(3), 507–526. 10.1037/met0000077 27266798 10.1037/met0000077 PMC5140785

[CR9] Bauer, D. J., & Hussong, A. M. (2009). Psychometric approaches for developing commensurate measures across independent studies: Traditional and new models. *Psychological Methods,**14*(2), 101–125. 10.1037/a0015583 19485624 10.1037/a0015583 PMC2780030

[CR10] *Bendler, J., & Reinecke, J. (2025). A tutorial on Bayesian multiple-group comparisons of latent growth curve models with count distributed variables. *Behavior Research Methods*, *57*, 112. 10.3758/s13428-025-02624-3 10.3758/s13428-025-02624-3 PMC11893654 40064801

[CR11] Boers, K., Reinecke, J., Seddig, D., & Mariotti, L. (2010). Explaining the development of adolescent violent delinquency. *European Journal of Criminology,**7*(6), 499–552. 10.1177/1477370810376572

[CR12] Bollen, K. A. (1989). Structural equations with latent variables. *John Wiley & Sons*. 10.1002/9781118619179

[CR13] Bollen, K. A., & Curran, P. J. (2006). *Latent curve models: A structural equation perspective*. Wiley.

[CR14] Brüderl, J., Drobnič, S., Hank, K., Neyer, F. J., Walper, S., Alt, P., Borschel, E., Bozoyan, C., Garrett, M., Geissler, S., Avilés, T. G., Gröpler, N., Hajek, K., Herzig, M., Lenke, R., Lorenz, R., Lutz, K., Peter, T., Preetz, R., ..., Wetzel, M. (2022). The German family panel (pairfam) (ZA5678 data file version 13.0.0). GESIS data archive. Cologne. 10.4232/pairfam.5678.13.0.0

[CR15] Cheung, G. W., Cooper-Thomas, H. D., Lau, R. S., & Wang, L. C. (2021). Testing moderation in business and psychological studies with latent moderated structural equations. *Journal of Business and Psychology,**36*(6), 1009–1033. 10.1007/s10869-020-09717-0

[CR16] Cortina, J. M., Markell-Goldstein, H. M., Green, J. P., & Chang, Y. (2021). How are we testing interactions in latent variable models? Surging forward or fighting shy? *Organizational Research Methods,**24*(1), 26–54. 10.1177/1094428119872531

[CR17] Cox, K., & Kelcey, B. (2021). Croon’s bias corrected estimation of latent interactions. *Structural Equation Modeling: A Multidisciplinary Journal,**28*(6), 863–874. 10.1080/10705511.2021.1922283

[CR18] Cox, K., Kelcey, B., & Bai, F. (2023). Croon’s bias-corrected estimation for multilevel structural equation models with latent interactions. *Structural Equation Modeling: A Multidisciplinary Journal,**30*(3), 467–480. 10.1080/10705511.2022.2140290

[CR19] *Cox, K., & Kelcey, B. (2025). Comparing Bayesian estimation and structural-after-measurement approaches for structural equation models with latent interactions and complex data structures. *Behavior Research Methods, 57*, 320. 10.3758/s13428-025-02840-x 10.3758/s13428-025-02840-x PMC12546545 41125847

[CR20] Croon, M. (2002). Using predicted latent scores in general latent structure models. In G. Marcoulides & I. Moustaki (Eds.), *Latent variable and latent structure modeling* (pp. 195–223). Erlbaum.

[CR21] Depaoli, S., Kaplan, D., & Winter, S. D. (2023). Foundations and extensions of Bayesian structural equation modeling. R.H. Hoyle (Ed.), *Handbook of structural equation modeling* (pp. 701–721). The Guilford Press.

[CR22] Dijkstra, T. K., & Schermelleh-Engel, K. (2014). Consistent partial least squares for nonlinear structural equation models. *Psychometrika,**79*(4), 585–604. 10.1007/s11336-013-9370-0 24306555 10.1007/s11336-013-9370-0

[CR23] Hayduk, L. A. (1987). *Structural equation modeling with LISREL: Essentials and advances.* Johns Hopkins University Press.

[CR24] Holtmann, J., & Koslowski, K. (2025). Modeling within-level latent interaction effects in multilevel vector-autoregressive models. *Behavior Research Methods,**57*, 277. 10.3758/s13428-025-02694-3 40913220 10.3758/s13428-025-02694-3 PMC12413427

[CR25] *Humberg, S., Grund, S., & Nestler, S. (2024). Estimating nonlinear effects of random slopes: A comparison of multilevel structural equation modeling with a two-step, a single-indicator, and a plausible values approach. *Behavior Research Methods*, *56*(7), 7912–7938. 10.3758/s13428-024-02462-9 10.3758/s13428-024-02462-9 PMC11362328 39060861

[CR26] *Irmer, J. P., Klein, A. G., & Schermelleh-Engel, K. (2024a). Model-implied simulation-based power estimation for correctly specified and distributionally misspecified models: Applications to nonlinear and linear structural equation models. *Behavior Research Methods*, *56*(8), 8955–8991. 10.3758/s13428-024-02507-z 10.3758/s13428-024-02507-z PMC11525309 39354129

[CR27] *Irmer, J. P., Klein, A. G., & Schermelleh-Engel, K. (2024b). Estimating power in complex nonlinear structural equation modeling including moderation effects: The powerNLSEM R-package. *Behavior Research Methods, 56*(8), 8897–8931. 10.3758/s13428-024-02476-3 10.3758/s13428-024-02476-3 PMC11525415 39304602

[CR28] Jongerling, J., Laurenceau, J. P., & Hamaker, E. L. (2015). A multilevel AR(1) model: Allowing for inter-individual differences in trait-scores, inertia, and innovation variance. *Multivariate Behavioral Research,**50*(3), 334–349. 10.1080/00273171.2014.1003772 26610033 10.1080/00273171.2014.1003772

[CR29] Jöreskog, K. G. (1971). Simultaneous factor analysis in several populations. *Psychometrika,**36*(4), 409–426. 10.1007/bf02291366

[CR30] Kelava, A., & Brandt, H. (2009). Estimation of nonlinear latent structural equation models using the extended unconstrained approach. *Review of Psychology,**16*(2), 123–132.

[CR31] *Kiefer, C., Wilker, S., & Mayer, A. (2024). Interactions between latent variables in count regression models. *Behavior Research Methods*, *56*(8), 8932–8954. 10.3758/s13428-024-02483-4 10.3758/s13428-024-02483-4 PMC11525413 39187739

[CR32] Klein, A. G., & Moosbrugger, H. (2000). Maximum likelihood estimation of latent interaction effects with the LMS method. *Psychometrika,**65*(4), 457–474. 10.1007/BF02296338

[CR33] Marsh, H. W., Wen, Z., & Hau, K.-T. (2004). Structural equation models of latent interactions: Evaluation of alternative estimation strategies and indicator construction. *Psychological Methods,**9*(3), 275–300. 10.1037/1082-989X.9.3.275 15355150 10.1037/1082-989X.9.3.275

[CR34] Marsh, H. W., Morin, A. J., Parker, P. D., & Kaur, G. (2014). Exploratory structural equation modeling: An integration of the best features of exploratory and confirmatory factor analysis. *Annual Review of Clinical Psychology,**10*(1), 85–110. 10.1146/annurev-clinpsy-032813-153700 24313568 10.1146/annurev-clinpsy-032813-153700

[CR35] Marsh, H., Guo, J., Dicke, T., Parker, P. D., & Craven, R. G. (2020). Confirmatory factor analysis (CFA), exploratory structural equation modeling (ESEM), and Set-ESEM: Optimal balance between goodness of fit and parsimony. *Multivariate Behavioral Research,**55*(1), 102–119. 10.1080/00273171.2019.1602503 31204844 10.1080/00273171.2019.1602503

[CR36] McNeish, D., & Hamaker, E. L. (2019). A primer on two-level dynamic structural equation models for intensive longitudinal data in Mplus. *Psychological Methods,**25*(5), 610–635. 10.1037/met0000250 31855015 10.1037/met0000250

[CR37] Mislevy, R. J. (1991). Randomization-based inference about latent variables from complex samples. *Psychometrika,**56*(2), 177–196. 10.1007/BF02294457

[CR38] *Muench, F. F., & Koch, T. (2025). Testing measurement and structural invariance in latent mediation models - A comparison of IPCR and Bayesian MNLFA. *Behavior Research Methods*, *57*, 250. 10.3758/s13428-025-02781-5 10.3758/s13428-025-02781-5 PMC12334378 40779090

[CR39] *Navarro, K. & Schermelleh-Engel, K. (2025). Cause for concern: Omitted cross-loadings in measurement models of nonlinear structural equation models. *Behavior Research Methods, 57*, 328. 10.3758/s13428-025-02792-2 10.3758/s13428-025-02792-2 41168605

[CR40] Nelder, J. A., & Wedderburn, R. W. M. (1972). Generalized linear models. *Journal of the Royal Statistical Society, Series A (General),**135*(3), 370. 10.2307/2344614

[CR41] Ng, J. C. K., & Chan, W. (2020). Latent moderation analysis: A factor score approach. *Structural Equation Modeling: A Multidisciplinary Journal,**27*(4), 629–648. 10.1080/10705511.2019.1664304

[CR42] Pokropek, A., Żółtak, T., Davidov, E., Meuleman, B., & Schmidt, P. (2025). *Challenges in multilevel modelling: Cross-group measurement noninvariance and measurement errors*. Sociological methods & research. Advance online publication. 10.1177/00491241251379459

[CR43] Rockwood, N. J. (2021). Efficient likelihood estimation of generalized structural equation models with a mix of normal and nonnormal responses. *Psychometrika,**86*(2), 642–667. 10.1007/s11336-021-09770-5 34091812 10.1007/s11336-021-09770-5

[CR44] Rosseel, Y. (2012). Lavaan: An R package for structural equation modeling. *Journal of Statistical Software,**48*(2), 1–36. 10.18637/jss.v048.i02

[CR45] Rosseel, Y., & Loh, W. W. (2024). A structural after measurement approach to structural equation modeling. *Psychological Methods,**29*(3), 561–588. 10.1037/met0000503 36355708 10.1037/met0000503

[CR46] *Rosseel, Y., Burghgraeve, E., Loh, W. W., & Schermelleh-Engel, K. (2025). Structural after measurement (SAM) approaches for accommodating latent quadratic and interaction effects. *Behavior Research Methods*, *57*, 101. 10.3758/s13428-024-02532-y 10.3758/s13428-024-02532-y 40009313

[CR47] *Schamberger, T., Schuberth, F., & Henseler, J. (2026). Moderated mediation with composites: The composite moderated structural equations approach. *Behavior Research Methods*. (In press). 10.3758/s13428-025-02930-w PMC13053372 41942788

[CR48] Schuberth, F. (2023). The Henseler-Ogasawara specification of composites in structural equation modeling: A tutorial. *Psychological Methods,**28*(4), 843–859. 10.1037/met0000432 34914475 10.1037/met0000432

[CR49] Smid, S. C., & Rosseel, Y. (2020). SEM with small samples: Two-step modeling and factor score regression versus Bayesian estimation with informative priors. In R. van de Schoot & M. Miocevic (Eds.), *Small sample size solutions: A guide for applied researchers and practitioners* (pp. 239–254). Routledge.

[CR50] Sörbom, D. (1974). A general method for studying differences in factor means and factor structure between groups. *British Journal of Mathematical and Statistical Psychology,**27*(2), 229–239. 10.1111/j.2044-8317.1974.tb00543.x

[CR51] Vehtari, A., Gelman, A., & Gabry, J. (2017). Practical Bayesian model evaluation using leave-one-out cross-validation and WAIC. *Statistics and Computing,**27*(5), 1413–1432. 10.1007/s11222-016-9696-4

[CR52] von Davier, M., Gonzalez, E., & Mislevy, R. (2009). What are plausible values and why are they useful? In M. von Davier & D. Hastedt (Eds.), *IERI monograph series: Issues and methodologies in large-scale assessments* (Vol. 2, pp. 9–36). IERI Institute/Educational Testing Service.

[CR53] Wall, M. M., & Amemiya, Y. (2000). Estimation for polynomial structural equation models. *Journal of the American Statistical Association,**95*(451), 929–940. 10.1080/01621459.2000.10474283

[CR54] Wall, M. M., & Amemiya, Y. (2003). A method of moments technique for fitting interaction effects in structural equation models. *British Journal of Mathematical and Statistical Psychology,**56*(1), 47–63. 10.1348/000711003321645331 12803821 10.1348/000711003321645331

